# A Systematic Review of Carotenoids in the Management of Age-Related Macular Degeneration

**DOI:** 10.3390/antiox10081255

**Published:** 2021-08-05

**Authors:** Drake W. Lem, Pinakin Gunvant Davey, Dennis L. Gierhart, Richard B. Rosen

**Affiliations:** 1 College of Optometry, Western University of Health Sciences, Pomona, CA 91766, USA; drake.lem@westernu.edu; 2 ZeaVision, LLC, Chesterfield, MO 63005, USA; dgierhart@zeavision.com; 3 Department of Ophthalmology, New York Eye and Ear Infirmary of Mount Sinai, Icahn School of Medicine at Mount Sinai, New York, NY 10029, USA; rrosen@nyee.edu

**Keywords:** carotenoids, macular pigment, macular pigment optical density, MPOD, lutein, zeaxanthin, *meso*-zeaxanthin, age-related macular degeneration, retinal neurodegeneration

## Abstract

Age-related macular degeneration (AMD) remains a leading cause of modifiable vision loss in older adults. Chronic oxidative injury and compromised antioxidant defenses represent essential drivers in the development of retinal neurodegeneration. Overwhelming free radical species formation results in mitochondrial dysfunction, as well as cellular and metabolic imbalance, which becomes exacerbated with increasing age. Thus, the depletion of systemic antioxidant capacity further proliferates oxidative stress in AMD-affected eyes, resulting in loss of photoreceptors, neuroinflammation, and ultimately atrophy within the retinal tissue. The aim of this systematic review is to examine the neuroprotective potential of the xanthophyll carotenoids lutein, zeaxanthin, and *meso*-zeaxanthin on retinal neurodegeneration for the purpose of adjunctive nutraceutical strategy in the management of AMD. A comprehensive literature review was performed to retrieve 55 eligible publications, using four database searches from PubMed, Embase, Cochrane Library, and the Web of Science. Epidemiology studies indicated an enhanced risk reduction against late AMD with greater dietary consumption of carotenoids, meanwhile greater concentrations in macular pigment demonstrated significant improvements in visual function among AMD patients. Collectively, evidence strongly suggests that carotenoid vitamin therapies offer remarkable synergic protection in the neurosensory retina, with the potential to serve as adjunctive nutraceutical therapy in the management of established AMD, albeit these benefits may vary among different stages of disease.

## 1. Introduction

Age-related macular degeneration (AMD) is the leading cause of irreversible blindness among older adults in developed countries, affecting roughly one in eight individuals aged 60 years or greater [1,2,3,4]. Comprehensive reports estimate that 200 million people currently live with AMD globally, with this aggregate expected to increase further and approach 300 million by 2040 [3]. Similar rising trends in the U.S. are expected to reach 5.4 million people by the year 2050 [1,4,5]. Although the majority of these are of Caucasian descent, the disease is not limited to individuals of Caucasian origin; Asian, Hispanic, and African-American populations tend to develop intermediate dry AMD and wet polypoidal choroidal vasculopathy with greater incidence [5,6,7]. The projected growth in prevalence among adults developing noncommunicable eye diseases, such as AMD, can be attributed to the demographic transition consistent with an aging global population [1,3,8,9,10]. Due to its chronic nature, wherein this incurable disease requires steady long-term management, AMD has become, and will remain, a public health challenge for both high- and low-income countries, with considerable socio-economic implications and rises in healthcare expenditures [1,2,3,8,9,10,11,12,13,14].

Aging remains one of the primary risk factors in AMD [1,4,9,15]. While cellular senescence is inherent to biological aging, these perturbations in photoreceptor cells and retinal pigment epithelium (RPE) are thought to bring about the neurodegenerative onset characteristic of age-onset maculopathy. Additional non-modifiable risk factors include sex, confirmed family history of AMD, and strong genetic factors that may further predispose individuals to this condition [1,5,12,16,17,18,19,20,21]. Genetic variants associated with complement factor H (CFH) and AMD susceptibility gene 2 (ARMS2) are well-established risk factors for the development and progression of AMD [16,17,18,19,20,21,22,23,24,25] (single nucleotide polymorphisms that are found on chromosomes 1q31 and 10q26, respectively). Conversely, modifiable risk factors for AMD include dietary behaviors, smoking status, cardiovascular disease, as well as metabolic comorbidities [5,26,27,28,29,30]. Among these, individuals who currently smoke (and past-smokers) carry significantly greater risk of incident AMD [26,27,28,31].

**Figure 1:**
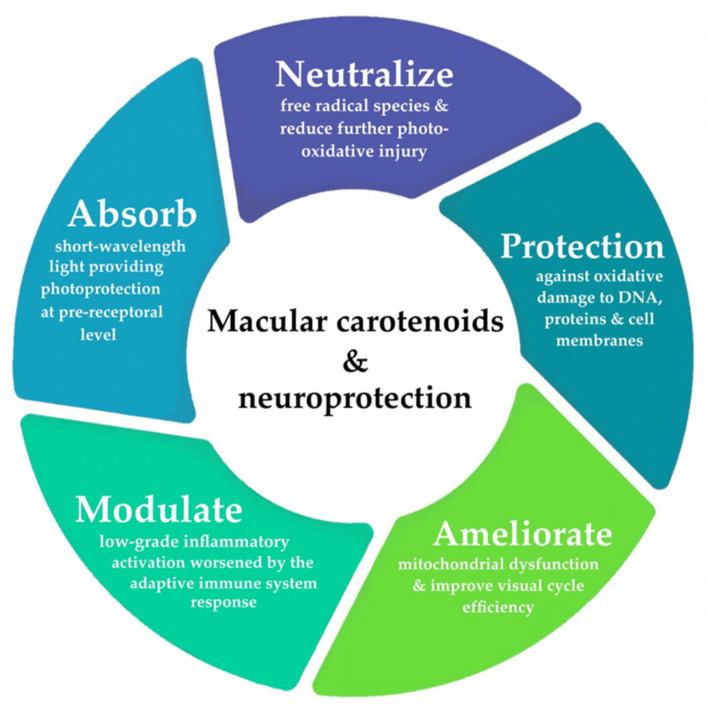
Overview of the neuroprotective mechanisms of xanthophylls lutein, zeaxanthin, and *meso*-zeaxanthin in the central retina.

The etiopathogenesis of AMD is complex and multifactorial. It is postulated that early and intermediate stages of maculopathy are predominated by oxidative stress and low-grade inflammatory activation in aging retinae [32,33,34,35,36,37,38,39,40,41]. Figure 1 provides a summary of the neuroprotective mechanisms provided by macular carotenoids. A comprehensive review of the precise molecular processes, by which carotenoids offer protection against photo-oxidative damage, has been discussed in detail elsewhere [42]. As a consequence of its extremely high metabolic activity and constant exposure to light, the outer retina is known to be particularly vulnerable to photo-oxidative injury and mitochondrial dysfunction, prompting the overproduction of free radical species [42,43,44,45,46]. A growing body of evidence implicates that compromised antioxidant capacity may also serve a crucial role in AMD pathology, a sequela, which occurs predominantly in response to the chronic cycles of sustained oxidative stress, paired with the concomitant depletion of endogenous antioxidants [42,43,46,47,48]. Local inhibition of these antioxidant defense mechanisms (to counteract the accumulation of toxic byproducts and cellular debris) plays a significant role in perpetuating subsequent neurodegenerative damage onto the surrounding tissues through immunostimulatory activity [35,40,41,43,46]. In fact, outer retinal lesions originating from oxidative insult have been shown to mediate a para-inflammatory state or an adaptive immune response to dysregulated complement activation [32,33,34,35,36,37,38,40,41,45,49]. This interdependence between cellular senescence and redox imbalance likely represents essential facets contributing to neurodegenerative onset and disease progression in AMD.

The body’s intrinsic homeostatic mechanisms for maintaining redox control are comprised of both exogenous and endogenous antioxidant activity for neutralizing free radical species [42,43,46,47,48]. The mainstay of preventative nutritional therapies is aimed at the augmentation of exogenous antioxidant defenses through oral supplementation containing nutraceuticals and micronutrients. In particular, xanthophyll carotenoids possess unique properties, serving as potent antioxidants and anti-inflammatory mediators in the retina, and have been demonstrated to benefit the prevention of neurodegenerative retinopathies, such as AMD and diabetic eye disease [42,43,50,51,52,53]. Greater dietary intake of xanthophylls, via carotenoid supplementation, has been well-documented to offer clinically meaningful benefits in visual performance, in both healthy and diseased states [54,55,56,57,58]. However, the comprehensive neuroprotective capacity, afforded by macular carotenoids in clinical management of AMD, has not been thoroughly discussed. Hence, the purpose of this systematic review concentrates on summarizing the available evidence from observational studies and randomized controlled trials, reporting on carotenoid lutein, *meso*-zeaxanthin, and zeaxanthin (only in patients with AMD).

### 1.1. Age-Related Macular Degeneration

Traditionally, color fundus photography and slit lamp biomicroscopy have been the mainstay for the ophthalmic examination of fundus lesions associated with AMD [15,59,60]. Several disease classification systems have been developed over the years, from population studies [61,62,63] and clinical-based trials, of which the Age-Related Eye Disease Study (AREDS) clinical severity scale (Table 1) and its simplified severity scale are most notable [64,65,66,67]. However, inconsistency in disease terminology and ambiguous definitions, relating to the severity of maculopathy, highlight an overwhelming unmet clinical need. Thus, it is highly recommended for all those working in this field to adopt the single diagnosis of AMD proposed by the Beckman classification (Table 2) [68], which is then further classified according to disease severity, in agreement with AREDS [64,65,66,67].

Retinal drusen and basal laminar deposits are sine qua non features of early AMD [69,70,71,72]. Drusen is formed by the exudative accumulation of various cellular waste products and atherogenic debris (>40% of drusen volume) [69,70,71,72,73]. Notably, the detection of subretinal drusenoid deposits above the RPE, also known as reticular pseudodrusen, are correlated with a two-fold increase in the risk of developing late-stage geographic atrophy [72,74]. Further retinal injury is evident by the presence of pigmentary changes (depigmentation or hyperpigmentation) found in the RPE, indicating abnormalities associated with intermediate AMD [15,70]. Often, these more preliminary stages of AMD do not carry obvious changes in visual function or may present elusive symptoms of mild distortion in central vision, with greater difficulty under low light conditions, such as reading or seeing at night [15,65]. Some reports suggest that dark adaptation dysfunction, marked by impaired recovery of light sensitivity mediated by rod photoreceptors under mesopic conditions, may be one of the earliest indications of AMD onset [75,76,77,78,79,80].

According to its pathophysiology, late AMD can be divided into neovascular AMD and geographic atrophy (GA), based on distinct clinical manifestations [15]. Neovascular proliferations are characterized in accordance with the compartment wherein the choroidal neovascularization complex occupies, and the formation of chorioretinal anastomoses is found upon fluorescein angiography. With the advent of optical coherence tomography (OCT) imaging modalities, the anatomical classification of neovascular subtypes is made possible, which includes: type 1 neovascularization (NV) for submacular lesions confined below the RPE, type 2 NV for subretinal lesions found in the space between the photoreceptor layer and RPE, and type 3 NV for intraretinal lesions found within the retinal layers [15,81,82,83,84,85,86,87,88,89]. Subsequently, the aberrant angiogenesis of fragile vasculature is often accompanied by the presence of a retinal hemorrhage, a hard exudate, detachments of the RPE, and chorioretinal fibrotic scarring [15,82,83,84,87,89,90]. On the other hand, non-neovascular complications, seen in GA, are characterized by the demarcated regions of hypopigmentation, in consequence of the cumulative degeneration of the outer neurosensory retina, RPE, and choriocapillaris [15,64,68,90,91,92]. Perilesional hyperpigmentation delineates areas of ongoing atrophy and the confluent superimposition of RPE cells [93,94,95]. Both forms of late AMD result in severe visual defects, such as the formation of central scotoma, while peripheral vision remains relatively intact. Neovascular AMD often develops quite rapidly, leading to acute vision loss within a relatively short period of time (days to months); meanwhile, atrophic lesions progress more gradually and may take years, or decades, for symptoms to manifest [15].

Given the increasing prevalence and substantial implications on quality of life, the detection of these phenotypic lesions in each stage of AMD is of profound importance for disease management and clinical screening. Thus, apposite surrogates of AMD pathology will likely serve a cardinal role in preventing the onset of extensive neurodegeneration within outer retinal layers and irreversible loss of photoreceptor cells.

### 1.2. Macular Pigment Optical Density in AMD—Background

The xanthophyll carotenoids lutein and zeaxanthin, as well as an isomer of lutein *meso*-zeaxanthin, serve an important role in sustaining the integrity of the retina concomitant with optimizing central visual acuity [42,96,97]. Collectively, these lipid-soluble carotenoids comprise the macular pigment, which forms a yellow spot that is seen during ophthalmoscopy. A recent imaging study determined that the spatial profiles of lutein and zeaxanthin are both localized in the fovea, as previously described [42,96,97,98,99,100]; however, only zeaxanthin was primarily concentrated in the inner plexiform (IPL), outer plexiform (OPL), and outer nuclear layers [98]. Meanwhile, lutein distribution was more dispersed throughout the macula, in reduced concentrations, when compared to foveal zeaxanthin levels [98]. Humans are unable to naturally synthesize lutein and zeaxanthin [97,101,102]; therefore, they must be acquired through the dietary consumption of foods, such as spinach, kale, and cruciferous green leafy vegetables, as well as corn and egg yolks [42,97,103,104]. On the other hand, *meso*-zeaxanthin is a biochemical isomer, also found in the macula, that is configured from lutein metabolism via RPE65 isomerase activity [105,106] within the retinal pigment epithelial cells [42,97,98,101,107]. A growing body of evidence indicates that the depletion of these carotenoids, marked by low macular pigment optical density (MPOD), may be a clinical biomarker associated with greater risk of incident retinopathy and visual dysfunction [42,43,108,109,110].

Numerous reports have shown clinical benefits, by raising the levels of xanthophylls in the retina through dietary supplementation, thus, adjunctive carotenoid vitamin therapy may offer enhanced neuroprotection by augmenting MPOD and subsequently preventing further injury [42,96,97,101,107,111,112,113,114,115,116,117,118,119,120,121,122]. Higher levels of MPOD are thought to preserve retinal tissue, specifically the layers containing photoreceptors in the fovea, through two primary mechanisms: (1) serving as an innate optical filter against blue light and (2) as protective antioxidants, by neutralizing free radicals and reducing consequent oxidative injury [97,103,108,112,123,124,125]. The peak wavelength of the absorption spectrum of the macular (~460 nm) attenuate proliferation of reactive oxygen species is generated by photosensitizers, such as rod and cone cells, exposed to a range of visible blue light (400–500 nm) [96,123,126]. This optical filtration is particularly significant, as short-wavelength (blue) light is highly reactive and has the capacity to exacerbate photo-oxidative degeneration in the most sensitive layers of the neurosensory retina [42,97,123,124,125,126,127].

### 1.3. Measuring MPOD

While several imaging techniques are used to measure MPOD non-invasively within optometry settings, each possess their own set of advantages and disadvantages. The abilities and shortcomings of the MPOD measuring techniques are outlined in more detail elsewhere [42,95,99,103,127,128,129,130,131,132,133,134,135,136]. In brief, the standard routine methods of heterochromatic flicker photometry (HFP) and customized flicker photometry (cHFP) [42,99,103,128,129,130,131] utilize a psychophysical approach, wherein the determination of macular pigment levels is reliant upon subjective participation [137,138,139]. Objective techniques of fundus reflectometry [114,140,141,142,143,144], autofluorescence (AFI) [95,132,134,135,145], and resonance Raman spectroscopy [98,146,147,148,149] collect MPOD measurements, utilizing physical properties of light within the retina [42,95,103,128,133,136,150].

### 1.4. MPOD Biomarkers in Clinical AMD

There is an overwhelming need for developing improved biomarkers that underscore the diverse pathology and subtypes found in patients with AMD. While current treatments have shown success for late neovascular AMD, there are a lack of proven therapies involving the mechanisms underlying early/intermediate stages and late atrophic stages of disease; in such cases, AMD develops, in consequence of the compounding cycles of oxidative stress and para-inflammation [151,152,153]. Therefore, it is critical that therapeutic targets are aimed at ameliorating the perturbations contributing to lesion formation and preventing irreversible retinal neurodegeneration. Biomarkers are important tools, with the capacity to significantly aid the development of novel therapeutics, in addition to investigating the efficacy and overall safety of available treatments [154,155,156,157]. Given that a single biomarker may be appropriate for different clinical utility, it is deemed necessary to clearly define the situation-specific context of how a particular biomarker will be used accordingly [154,155,156].

While advancements in multimodality imaging have improved the prognosis for diagnosing retinal abnormalities, these modalities have also enabled the measurement of macular pigment status, to serve as a biomarker in multiple settings for AMD. It has been well-documented that MPOD levels are substantially reduced in AMD patients [107,110,158,159,160,161,162,163,164], which may be explained, at least in part, to similar risk factors shared between them [5,21,31,107,110,158,159,160,161,162,163,164,165,166,167,168]. Diagnostic assessment, incorporating MPOD measurements, in conjunction with standard fundoscopic imaging, may offer unique clinical insight into the current state of the individual’s retinal health. In fact, macular pigment levels represent the local equilibrium between pro-oxidant stressors and antioxidant defenses in the retina, which can be attributed to its slow biological turnover [42]. To this accord, MPOD measurement may function as: (1) a prognostic biomarker to appraise the health of neuroretinal layers, (2) a susceptibility/risk biomarker for screening those at risk of incident AMD, and (3) a pharmacodynamic/response biomarker to determine the clinical benefits of carotenoid vitamin therapy in AMD.

As a prognostic biomarker, MPOD levels may be used to monitor the progression of neurodegenerative changes in the photoreceptors and ganglion cells among patients with early or intermediate AMD. One study found that macular pigment levels were positively correlated with central retinal thickness, along with the neural volume of the ganglion cell layer (GCL), inner plexiform layer, and outer nuclear layer [169]. Previous reports have demonstrated differential morphology changes on OCT within the outer retinal layers in patients with early AMD, including the thickness and volume of the photoreceptor layer, as well as the RPE-Bruch’s membrane complex [71,92,170,171,172,173,174,175]. Similarly, inner retinal alterations are also found in the macular ganglion cell complex, comprised of the IPL, ganglion cell layer, and nerve fiber layer [175,176,177,178,179,180], which correspond to the dendrites, cell bodies, and axons of the neurosensory ganglion cells, respectively. Thus, MPOD depletion may serve to help prognosticate visual outcomes before severe impairment develops in early/intermediate AMD patients.

Given its bilateral nature, AMD fellow eyes may be considered to represent the pre-disease condition, in the absence of early retinal lesions, based on the incidence of fellow eye involvement, which increases significantly over time [158,159,181,182]. Recently, Nagai et al. determined the risk of late AMD fellow eyes developing incident maculopathy was significantly associated with the combination of reduced MPOD (<0.65 density units (DU), measured by HFP) and photoreceptor outer segment length (<35 μm on OCT) [183]. These results suggest MPOD screening may be an important susceptibility/risk biomarker used for the early detection of subclinical neurodegeneration among older adults and eyes with greater risk of developing AMD.

Furthermore, serial measurement of MPOD is used as pharmacodynamic/response biomarkers in randomized clinical trials to evaluate the protective benefits of carotenoid supplementation in patients with AMD, as discussed in more detail below. In summary, MPOD levels could be used to function as susceptibility/risk, monitoring, and pharmacodynamic/response biomarkers, in accordance with FDA-NIH guidelines [154,155].

## 2. Materials and Methods

This systematic review was conducted in accordance with the reporting guidelines outlined by the Preferred Reporting Items for Systematic Reviews and Meta-Analysis (PRISMA) [184]. The study was registered on PROSPERO (registration number: 269373).

### 2.1. Literature Search and Selection Strategy

We conducted a comprehensive literature review in PubMed, Embase, the Cochrane Library, and the Web of Science, utilizing a combination of the follow keywords and their variations in the search query: lutein, zeaxanthin, *meso*-zeaxanthin, carotenoids, macular pigment, macular pigment optical density, MPOD, age-related macular degeneration, AMD, and neurodegeneration. We extracted all relevant publications from clinical studies that published data on the relationship between MPOD/carotenoids (including lutein, and/or zeaxanthin, and/or *meso*-zeaxanthin) and AMD, prior to 25 July 2021. Records retrieved from the initial search were vetted individually, by two authors (PGD and DWL), for proper selection criteria and then further examined using English titles and abstracts. We carefully searched through the qualifying publications’ reference lists and cited references to ensure that all relevant material was included in this review. All eligible, full-text publications were individually examined, by the same two authors (PGD and DWL), for the appropriate inclusion and exclusion criteria, discussed below; any disagreements were resolved through discussions with the third and fourth authors (DLG and RBR).

### 2.2. Study Selection Criteria

Observational clinical studies that adhered to the following criteria were included in this review: (1) epidemiological studies examining the association between the risk of incident AMD (including early or late AMD) and xanthophyll carotenoids, measured via dietary intake/consumption, serum concentrations, and/or by validated technique to quantify macular pigment optical density (MPOD) levels; (2) studies including adults with or without established AMD; and (3) original peer-reviewed publications available in English.

Prospective randomized clinical trials included in this review satisfied the following criteria: (1) interventional trials, investigating the effects of carotenoid vitamin therapy containing lutein, and/or zeaxanthin, and/or *meso*-zeaxanthin on clinical outcomes in eyes with AMD; (2) studies reporting on xanthophyll carotenoid levels in serum and/or by validated MPOD technique; (3) studies reporting on visual outcome measures, in response to carotenoid treatment; (4) must include adults with the presence of AMD; and (5) peer-reviewed, original research.

### 2.3. Data Extraction, Reliability and Risk of Bias Assessment

The authors carefully followed the PRISMA reporting guidelines, as closely as possible, as outlined above [184]. The risk of bias was assessed independently, by two authors (PGD and DWL), using the standardized metrics established to evaluate the interventional studies included in the present review. We used the Cochrane Collaboration’s tool for the assessment of risk of bias for randomized trials, which consists the following domains: selection bias, performance bias, detection bias, attrition bias, reporting bias, and other sources of bias [185].

## 3. Results

### 3.1. Search and Selection of Eligible Studies

**Figure 2:**
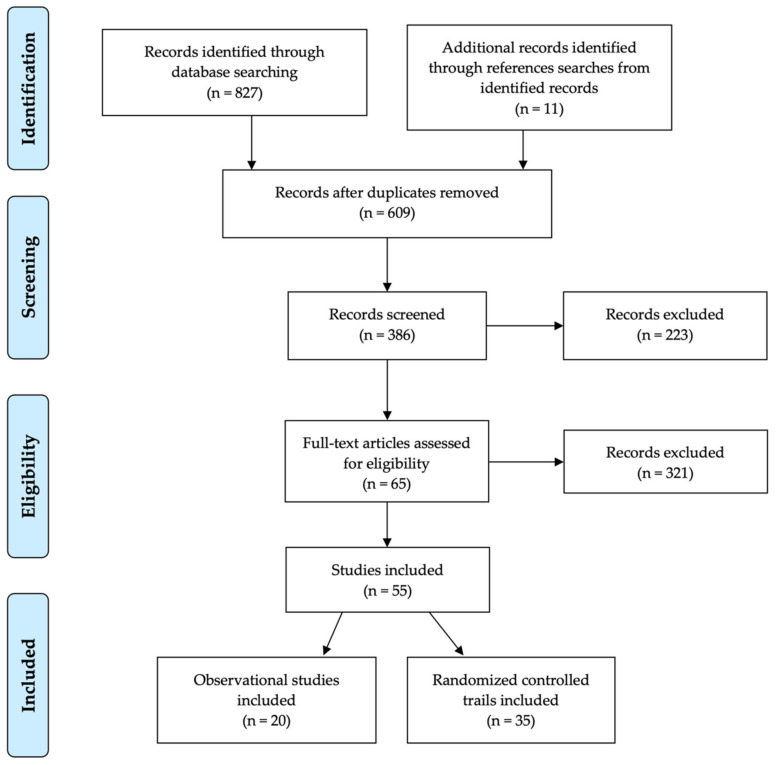
PRISMA flow chart, outlining the systematic review of xanthophyll carotenoids in the management of AMD.

In total, 827 entries were collected from the primary search, using only scientific databases. After removing the duplicate entries and retrieving 11 additional records from the reference searches, a total of 609 studies remained for the further screening of titles and abstracts. From these, 223 entries were eliminated for further screening, based on the manuscript type, with an additional 321 records being excluded, in accordance with the inclusion/exclusion criteria described above. Finally, 65 eligible publications were selected for full-text assessment, of which 55 studies were included in the final evaluation (Figure 2): 20 observational clinical studies [16,51,109,186,187,188,189,190,191,192,193,194,195,196,197,198,199,200,201,202] and 35 interventional clinical trials [50,53,115,117,119,120,203,204,205,206,207,208,209,210,211,212,213,214,215,216,217,218,219,220,221,222,223,224,225,226,227,228,229,230,231].

### 3.2. Carotenoids and Risk of AMD (Observational Studies)

Currently, dietary modifications remain the mainstay of therapeutic strategies, to potentially delay or prevent both the development and progression of AMD. The Age-Related Eye Disease Study (AREDS) is considered to be among the most influential large-scale clinical trials highlighting the relationship between dietary antioxidants and the risk of AMD progression [181]. Reports indicate that regular consumption of the AREDS micronutrient formula (containing vitamin C, vitamin E, beta-carotene, and zinc) offered modest benefits, reducing the risk of late AMD progression by up to 25% during a five-year follow-up with at risk patients [181]. In aging retinae, it is believed that the depletion of endogenous and exogenous antioxidants represents a critical driver in exacerbating neurodegenerative mechanisms. In fact, there is substantial evidence in favor of the neuroprotective association, between greater dietary consumption of carotenoid nutraceuticals, increased lutein and zeaxanthin concentrations in serum, and AMD prevention. A summary of these observational epidemiology studies is outlined in Table 3.

It is evident that the relationship between dietary behaviors, lifestyle choices, and the risk of AMD is complex and multifaceted, wherein unhealthy lifestyles often carry an increased risk of consequent disease [232]. Among older adults, early lifestyle modifications for the systemic management of metabolic syndrome is vital for slowing disease progression, as it also represents another risk factor [65,233]. Additionally, the National Eye Institute encourages greater consumption of leafy green vegetables to lower AMD risk [1]. Multiple longitudinal cohort studies have demonstrated greater consumption of foods such as spinach, kale, and collard greens on a regular basis carry significant protection against incident late AMD [51,187,194,196,197,199]. In fact, these green leafy vegetables, from the cruciferous *Brassica oleracea* cabbage species, are recognized as an excellent sources of xanthophyll carotenoids, lutein and zeaxanthin [197,234,235,236,237]. A meta-analysis by Ma et al. found that individuals with the highest levels of carotenoid intake saw a significant reduction in the risk of late AMD (pooled relative risk (RR) = 0.74; 95% confidence interval (CI): 0.57–0.97) and saw a 32% risk reduction for neovascular AMD (RR = 0.68; 95% CI: 0.51–0.92) [238]. Using data from the AREDS cohort, the calculated odds ratios (OR) from one case-control study seemed to corroborate these findings, when comparing the highest versus lowest quintiles of carotenoid intake. Greater dietary consumption of lutein and zeaxanthin offered an enhanced protection against neovascular AMD (OR = 0.65; 95% CI: 0.45–0.93), geographic atrophy (OR = 0.45; 95% CI: 0.24–0.86), and large, or extensive, intermediate drusen (OR = 0.73; 95% CI: 0.56–0.96) [51]. Evidence from a large cohort of studies largely seemed to implicate that the protective benefits of greater carotenoid intake may be confined to late AMD. However, one report, from the population-based Rotterdam Study, found that higher dietary antioxidants (including lutein and zeaxanthin) may significantly attenuate early AMD incidence conferred by genetic risk variants [16,25]. These results are encouraging, given that the AMD risk, among carriers of the *CFH Y402H* variant, increased by up to 11-fold, and those with the ARMS2 (*LOC387715 A69S*) variant carried up to 15-times greater risk [16,17,18,19,20,21,22,23,24,25]. One school of thought suggests that xanthophyll carotenoids afford synergistic neuroprotection against these risk alleles by limiting the overactivation of the complement system concomitant with mitochondriotropic augmentation, respectively [239,240,241,242]. Furthermore, these findings highlight the importance of stable dietary behaviors, involving the frequent consumption of nutraceuticals rich in lutein and zeaxanthin, for the management of established AMD.

Observational studies investigating the relationship between the serum levels of macular carotenoids provide some evidence of the protective benefits against age-related maculopathy [109,190,191,192]. The 1993 Eye Disease Case-Control Study first reported that greater levels of lutein and zeaxanthin in serum were inversely associated with the risk of neovascular AMD [191]. However, a 1995 analysis from the Beaver Dam Eye Study was unable to reproduce these findings and did not find serum levels to correlate with late AMD prevention [193]. Variation among these initial reports may be explained, at least in part, by differences in the ethnogeography of the sample, and sample size, whereby influencing the interpretability of these results. Moreover, serum analyses from population-based cohorts in the United Kingdom, France, and China seemed to corroborate the protective association with systemic increases of lutein and zeaxanthin concentrations in circulation [109,190,192]. Surprisingly, two of these studies illustrated that the serum levels of zeaxanthin were strongly associated with the risk of incident AMD (both early and late AMD) [109,192]. It is well known that differential dietary habits have significant implications on their absorption from food matrices, as well as subsequent concentrations within the plasma [235,243,244,245,246,247,248,249,250], and may, therefore, account for some of the inconsistency among reports.

It is important to note that similar biological mechanisms, which greatly reduce the bioavailability of lutein and zeaxanthin, are also involved with established AMD risk factors. The cumulative effect of compromised antioxidant capacity, in consequence of prolonged oxidative injury, is thought to create an overwhelming, neurodegenerative environment. Mitochondrial dysfunction and photo-oxidation are known to trigger the proliferation of premature cellular senescence in RPE, which subsequently triggers the pathogenic cascade of AMD development [32,38,49,239,240,241,242,251,252,253,254,255,256,257,258,259,260,261,262]. Moreover, in diabetic retinopathy, the underlying causes of metabolic syndrome have been shown to substantially compromise the assimilation and transport of dietary carotenoids [30,43,263,264,265,266,267,268,269,270,271,272,273,274,275,276,277,278,279,280,281,282]. Metabolic perturbations, such as obesity, insulin resistance, and chronic hyperglycemia promote atherogenic metabolic imbalance, which further contributes to macular pigment depletion [102,250,276,277,278,279,280,281,282,283,284,285]. Therefore, low MPOD levels likely represent an essential factor in AMD development.

Conversely, some observational studies were unable to confirm these benefits [186,187,189,193,195,201]. For instance, the Beaver Dam Eye Study found that neither dietary intake nor serum carotenoid levels were significantly correlated with AMD [193,201], while the Muenster Aging and Retina Study (MARS), in Germany, also observed a non-statistically significant association between plasma concentrations and age-related maculopathy [189]. Inconsistency among large-scale cohort studies may be attributed, at least in part, to the persistent challenges of investigating insidious neurodegenerative conditions, such as AMD. Etiologically relevant exposures involve a combination of lifestyle choices and dietary habits, culminating over years or decades before the date of diagnosis, as clinical manifestations often present themselves only after incurring extensive damage to the retina. Thirteen out of twenty (13/20) reports from several large-scale epidemiological studies demonstrated the effects of xanthophylls in protecting against the progression of AMD [16,51,109,187,190,191,192,194,196,197,198,199,202]. Thus, it is appropriate to summarize that the majority of the observational studies discussed herein advocate the benefits of xanthophyll carotenoids in AMD.

### 3.3. Carotenoids in the Management of AMD (Interventional Studies)

Given the effectiveness of the AREDS supplement in slowing the course of AMD progression, randomized clinical trials have investigated the efficacy of carotenoid vitamin therapy, supplemented with or without additional antioxidants and micronutrients. It is important to note that the original AREDS formulation did not include xanthophyll carotenoids (i.e., lutein or zeaxanthin) [65]; instead, it contained β-carotene, which belongs to the subclass of provitamin A carotenes [286]. However, in response to the discovery that β-carotene may correlate with a greater risk of developing lung cancer among cigarette smokers [287,288], the Age-Related Eye Disease Study 2 (AREDS2) modified the original formula by removing β-carotene and substituting with lutein and zeaxanthin [50,289]. Primary analysis from the AREDS2 trial suggested xanthophyll supplementation did not offer further benefits against the rate of AMD progression, in comparison to the original AREDS formula [50]. However, secondary analysis showed that lutein and zeaxanthin supplementation significantly improved protection against late AMD (hazard ratio (HR): 0.82; 95% CI: 0.69–0.96) and particularly against neovascular AMD (HR: 0.78; 95% CI: 0.64–0.94), when substituted for β-carotene in the AREDS formulation [203]. The risk reduction was most significant among those with intermediate AMD lesions (bilateral large drusen) at baseline; direct comparisons showed HRs of 0.76 (95% CI: 0.61–0.96; *p* = 0.02) for developing late AMD and 0.65 (95% CI: 0.49–0.85; *p* = 0.002) for neovascular AMD [203]. From these reports, the AREDS2 suggests that supplementation with lutein and zeaxanthin could offer enhanced protection and are well-suited for therapeutic management using nutraceuticals in patients with established AMD, particularly in lieu of β-carotene.

**Figure 3:**
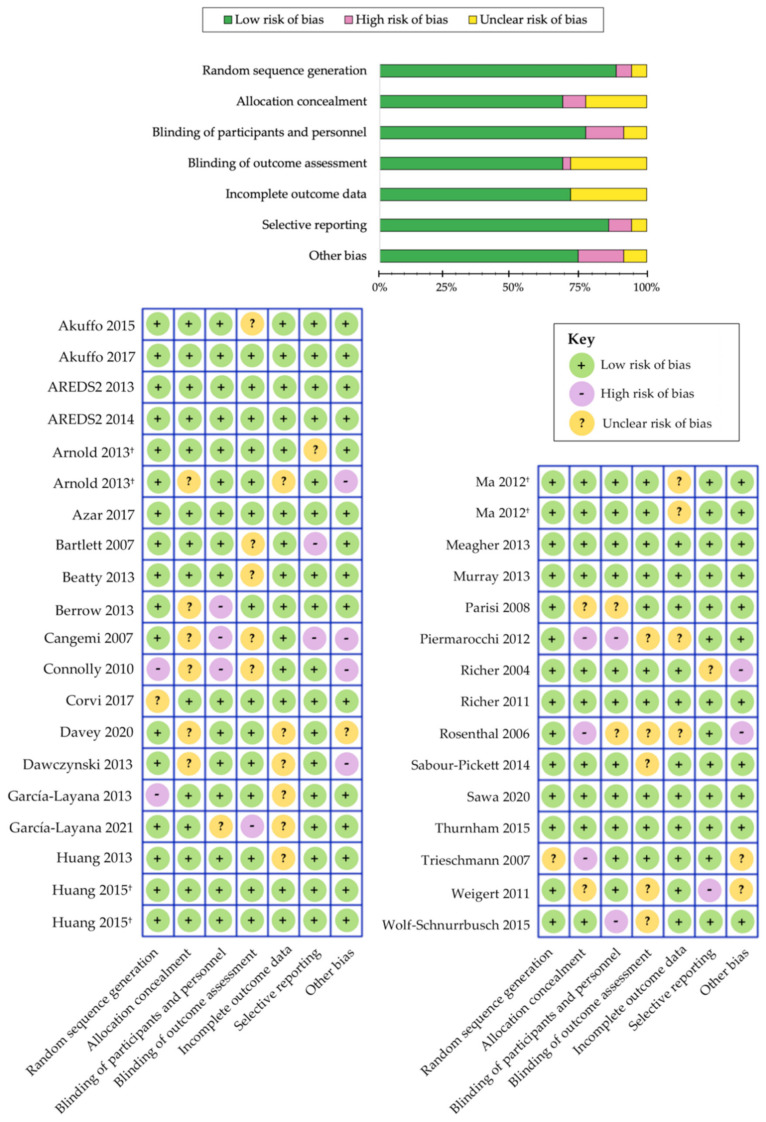
Cochrane Collaboration’s tool for assessing risk of bias in randomized controlled trials [185]. Separate publications are indicated with a symbol (†) next to author name.

Numerous randomized clinical trials have been extremely consistent in demonstrating that xanthophyll carotenoid supplementation can greatly improve their concentrations in serum [50,115,119,204,205,206,207,213,217,219,221,222,227,229,231] and within the retinal tissue (i.e., MPOD) among patients with AMD [56,115,117,119,120,204,205,206,210,213,214,215,216,222,225,226,228,230,231]. A summary of these randomized clinical trials is outlined in Table 4 and Table 5. We have evaluated the risk of bias among the various randomized controlled trials using the Cochrane Collaboration’s tool [185] and a summary is shown in Figure 3. Overall, we determined that the risk of bias was low among the randomized controlled trials that evaluated the benefits of carotenoid vitamin therapy in AMD. A meta-analysis by Ma et al., comparing nine carotenoid interventional trials, revealed a dose-response relationship that was positively correlated with increased MPOD levels and changes in plasma concentrations, following supplementation with lutein, zeaxanthin, and/or *meso*-zeaxanthin [56]. Stratified analysis demonstrated that the augmentation of the macular pigment was most effective when supplementing with all three xanthophyll carotenoids during trials lasting longer than 12 months [56]. A stronger effect was also noted for studies containing higher doses of these carotenoids (per daily serving). Furthermore, reports from similar clinical trials seem to corroborate these findings, wherein treatment with macular carotenoids offered significant improvements to MPOD levels in eyes with AMD, upon measurement with both subjective and objective techniques [115,117,205,210,213,214,226,228,230,231]. Consistency among these results is highly significant because changes in the macular pigment measured, in response to carotenoid vitamin therapy, substantiate the role of MPOD status in representing a pharmacodynamic/response biomarker in the context of AMD.

Based on the functional relationship between the macular pigments and sharp central vision, alterations in MPOD status have been postulated as a surrogate of visual performance in both healthy and diseased states [42]. In fact, a recent systematic review and meta-analysis by Johnson et al. found that MPOD was significantly correlated with visual function outcomes, including visual acuity, contrast sensitivity, photostress recovery, glare discomfort/disability, and dark adaptation in adults with healthy eyes [54]. Prior reports have also shown that improvements in visual function are positively associated with greater macular pigment levels [54,55,290,291,292,293,294,295,296,297,298,299], which can also be achieved through carotenoid supplementation in healthy eyes [55,57,58,111,112,114,116,298,299,300,301,302,303,304,305,306]. Thus, evidence from AMD trials, wherein carotenoid vitamin therapy is found to enrich MPOD concentrations concomitant with improvements in visual outcome measures, may be clinically beneficial for older adults and those with AMD, as it will likely render daily activities, such as reading or watching television, safer and easier [55,292].

In a meta-analysis by Liu et al. comparing data from several randomized, double-blind, placebo-controlled trials found that carotenoid supplementation resulted in significant improvements in best-corrected visual acuity (BCVA) and contrast sensitivity (CS) at all spatial frequencies in a dose-response relationship [307]. Correlation analysis revealed a linear association between the augmentation of MPOD levels and the observed benefits in BCVA. Several reports seem to mirror these findings, wherein xanthophyll carotenoids notably increased BCVA scores when supplemented for 12 months or longer [210,212,215,224,225,226]. Interestingly, Liu et al. noted that the magnitude of improvement in visual acuity among those with late AMD was substantially reduced, in comparison to those seen in eyes with early or intermediate AMD [307]. These findings likely underscore the mechanisms of action and criticality of the potential of xanthophyll carotenoids in ameliorating the integrity of the neurosensory retina before permanent loss of macular photoreceptors.

Although these improvements in visual acuity are encouraging, changes in CS function represent more comprehensive outcome measures for detecting subtle alterations in visual capacity, following treatment with carotenoid vitamin therapy. Indeed, CS is a more reliable measure of visual function that captures the essence of spatial visual sensitivity and is often prognosticative of poor visual performance, especially in eyes with maculopathy [308,309,310]. Furthermore, a significant linear association was shown between the positive changes in MPOD and the effects on CS at middle frequency [307]. In concordance with this meta-analysis, many AMD trials have also reported demonstrable improvements in CS at low and middle spatial frequencies, following significant enhancement in the macular pigment [53,115,117,204,205,224,225,226,228,231]. Similar to other reports, one study found that, in contrast to the considerable rise in MPOD from baseline to 24 weeks, statistically significant changes in CS were only observed after 48 weeks of supplementation [115]. Hence, these findings seem to suggest that MPOD status may represent a sine qua non for visual function improvement; for instance, contrast sensitivity would show significant improvement, only after MPOD had been sustained at greater concentrations [57,115,292]. This hypothesis is supported by the functional capacity of the macular pigments, wherein the preferential absorption of short-wavelength (blue) light provides pre-receptoral filtration, in addition to limiting the adverse effects of chromatic aberration [55,290,291,292,293,294,295,296,297,298,299]. Greater MPOD levels may also account for the improvements in glare disability [225,226] and photostress recovery [115,204,225]. Hence, carotenoid vitamin therapy was shown to significantly ameliorate several measures of visual performance that worsen, with respect to age and in patients with early or late AMD.

Several AMD trials also demonstrated remarkable improvements in objective measurement of macular function, following supplementation with xanthophyll carotenoids for twelve months or more [211,218,220,223]. Previous studies suggest that the functional integrity of the central retina, particularly the macular region, may be compromised during the early stages of disease progression [311,312,313,314,315,316,317,318,319,320,321]. Reports from AMD trials found that carotenoid vitamin therapy offered protection against early functional abnormalities within the central retina (between 0° and 5° eccentricity) [211,218,220,223]. In fact, two reports indicate that the improvements in central retinal function were positively correlated with MPOD augmentation [218,220]. These results may also be attributed, at least in part, to the enhanced neuroprotective capacity, afforded by these dietary antioxidants, to ameliorate pro-oxidative and pro-inflammatory mechanisms in the local tissue, particularly within the neurosensory layers of the macula [46,255,322,323,324]. In addition to improving the total antioxidant capacity, xanthophylls may also promote metabolic efficiency of the visual transduction cascade by augmenting mitochondrial dysfunction, a primary source of intracellular free radical formation in aging retina [42,46,47,48,255,298,324]. It has also been postulated that greater levels of carotenoids may help to promote the maintenance of synaptic network activity by enhancing cell survival and the viability of neurosensory cells [47,48,298]. However, additional studies are needed to further elucidate the potential role of the carotenoids involved with synaptic network activity and cognitive function [298,325]. These findings suggest that long-term treatment with carotenoids lutein and zeaxanthin in patients with AMD may promote enhanced retinal function by increasing macular pigment concentrations.

In summary, 21 randomized clinical trials reported on the efficacy of carotenoid vitamin therapy on augmenting MPOD levels (Table 5), of which 18 studies demonstrated statistically significant improvements [53,115,117,119,120,204,205,206,208,210,213,214,215,216,222,225,226,228,229,230,231]. Similarly, all 15 studies, highlighted in Table 4, saw demonstrable improvements in the serum concentrations of these xanthophylls, following oral supplementation [50,115,119,204,205,206,207,213,217,219,221,222,227,229,231]. Differential changes in visual performance measures were investigated among 18 studies reporting on visual acuity [50,53,115,117,120,205,208,210,211,212,214,215,216,217,222,224,225,226] and 15 studies reporting on contrast sensitivity function [53,115,117,204,205,209,210,211,216,224,225,226,228,229,231]. Improvements in BCVA were seen in six out of eighteen (6/18) trials [210,212,215,224,225,226]; meanwhile, remarkable benefits in CS were demonstrated in ten out of fifteen (10/15) randomized controlled trials [53,115,117,204,205,224,225,226,228,231]. Five studies evaluated changes in glare disability [204,209,225,226,229], of which four reports indicated significant improvement with carotenoid vitamin therapy [204,209,225,226]. Similarly, five clinical trials investigated the effect on photostress recovery time in AMD patients [115,117,204,225,226], wherein three reports saw changes of statistical significance [204,225,226]. Furthermore, each of the four studies, investigating the objective measures of retinal function through multifocal electroretinogram, showed significant improvements with carotenoid supplementation [211,218,220,223]. It is noteworthy to point out, as summarized in Table 5, that various randomized clinical trials demonstrated significant benefits of carotenoid vitamin supplementation in all stages of AMD.

Reports seem to indicate that AMD patients would likely require a minimum of twelve months of using carotenoid vitamin therapy and a higher dose of carotenoids before measurable benefits in visual function would become clinically apparent. The Carotenoids in Age-Related Maculopathy Italian Study (CARMIS) found that the relative risk of three or more letter visual loss was reduced by up to 76% (RR: 0.26; 95% CI: 0.11–0.59) among patients with atrophic AMD, following two years of carotenoid vitamin therapy [224]. Repeated measures analysis also demonstrated remarkable time effects were seen for the improvements in CS at 6, 12, and 24 months in the active treatment group [224]. This may explain, at least in part, why some trials with shorter durations reported increases in MPOD but only saw trends of improvement in visual function that did not achieve statistical significance [117,120,214,216,217,222]. However, more recent studies investigating the different ratios of xanthophyll carotenoids in formulation, namely the addition of *meso*-zeaxanthin, have largely shown that incorporating all three carotenoids may offer advantages for the management of early AMD [53,205,228,230].

It is important to note several potential limitations to these studies. In general, the consumption of any single micronutrient-containing vitamin does not appear to afford protection against AMD onset. Although, based on the current evidence, when combined with other antioxidants, dietary carotenoid supplementation with lutein, zeaxanthin, and/or *meso*-zeaxanthin does appear to substantially delay the disease progression in established AMD. The studies that looked at the addition of *meso*-zeaxanthin to the carotenoid formulation did not explore a separate group with *meso*-zeaxanthin alone. So, the exact benefit of including *meso*-zeaxanthin is not fully understood, as lutein should theoretically be converted to *meso*-zeaxanthin in all individuals that have the RPE65 isomerase. It is unknown (and additional research is needed) if greater amounts of lutein or zeaxanthin may be a sufficient and suitable substitute to *meso*-zeaxanthin or if *meso*-zeaxanthin is truly needed. It is noteworthy that xanthophyll carotenoids, plus antioxidants, did not exert similar treatment effects on geographic atrophy progression during the five-year follow-up in AREDS2 [203]. This may be attributed, at least in part, to advanced stages of disease and by poor micronutrient absorption rates, which likely represents a limiting factor and should not be ruled out from clinical trials [122,326]. However, one preliminary report indicated that oral zeaxanthin supplementation, as an adjunct to an aggressive triple combination therapy regimen (including bevacizumab, steroid, and photodynamic therapy with verteporfin) for patients with subfoveal choroidal neovascularization, enhanced therapeutic efficiency and decreased the number of treatment cycles required [327]. Similar benefits were reported in cultured human RPE cells, following hypoxia-induced VEGF secretion, whereby treatment with zeaxanthin was suggested to offer direction protection against the pro-angiogenic factors contributing to neovascular lesions [328]. Thus, improving carotenoid bioavailability should be among the primary aims for future interventional trials. The bioavailability of carotenoids, following assimilation and transport from dietary matrices, is also strongly influenced by age, gender, and ethnic origin, as well as anthropometric characteristics [43,47,250,266,270,275,277,278,279,281,329]. To overcome such limitations, advancements in micronized and nanoemulsion-based micronutrient delivery techniques have demonstrated improved bioavailability and accumulation of xanthophyll carotenoids in the retina, while maintaining overall safety [53,122,270,330,331,332]. Also, the measurement of MPOD longitudinally can provide a measure of “true bioavailability” at the end organ, which is targeted by the carotenoid vitamin therapy.

## 4. Conclusions

Age-related macular degeneration remains a leading cause of modifiable vision loss in older adults within developed countries. There is a strong rationale for the use of adjunctive carotenoid vitamin therapy in the clinical management strategies for established AMD. It appears that in the aging retina, the inhibition of the endogenous antioxidant capacity, marked by the depletion of macular carotenoids lutein, zeaxanthin, and *meso*-zeaxanthin, is a major contributing factor in disease progression. Additionally, systemic perturbations, associated with metabolic syndrome, inversely impact the bioavailability and localization of these carotenoids to the retina. It has been well-documented that these xanthophyll carotenoids possess profound antioxidant capacity, concomitant with their ability to serve as anti-inflammatory mediators, thereby limiting subsequent injury to surrounding tissues. Consistent evidence from large-scale epidemiology studies, and several randomized clinical trials, substantiate the synergic neuroprotective benefits afforded by carotenoid vitamin therapy in eyes with any stage of AMD. To this accord, the routine measurement of MPOD levels may be appropriate for monitoring retinal neurodegeneration caused by AMD and screening those at high-risk prior to the onset of clinical lesions associated with maculopathy. There is tremendous need for timely and prophylactic treatment strategies, whereby irreversible vision loss can be delayed or prevented. Hence, carotenoid vitamin therapy shows enhanced neuroprotection and clinical benefits in visual function, as an adjunctive nutraceutical strategy for patients with AMD.

## Figures and Tables

**Table 1:** AREDS clinical severity scale of age-related macular degeneration (AMD) [65].

Category	Age-Related Eye Disease Study (AREDS) Classification
1	No drusen, or non-extensive small drusen only in both eyes
2	Extensive small drusen, non-extensive intermediate drusen, or the presence of pigment abnormalities in at least one eye
3	Extensive intermediate drusen, large drusen, or non-central geographic atrophy in at least one eye
4	Advanced AMD as defined by at least one of the following: geographic atrophy, retinal pigment epithelial detachment in one eye, choroidal neovascularization, or scars of confluent photocoagulation; or visual acuity less than 20/32 associated with lesions from non-advanced age-related macular degeneration, including large drusen in the fovea, in only one eye

Abbreviations: AMD, age-related macular degeneration.

**Table 2:** Beckman Classification System of AMD.

Beckman Clinical Classification [68]			
AMD Classification ^1^	Drusen	Pigmentary Abnormalities ^2^	Additional Features
No apparent aging	None	None	n/a
Normal aging changes	Small (≤63 μm)	None	n/a
Early AMD	Medium (>63 μm and ≤125 μm)	None	n/a
Intermediate AMD	Large (>125 μm)	Abnormalities present ^2^	n/a
Late AMD	Large (>125 μm)	Abnormalities present ^2^	Neovascular AMD and/or any geographic atrophy

^1^ AMD classification based on lesions, assessed within 2 disc diameters of the fovea in either eye. ^2^ AMD pigmentary abnormalities defined as any hyper- or hypo-pigmentary abnormalities associated with at least some medium drusen but not associated with known disease entities. Abbreviations: AMD, age-related macular degeneration; n/a: not applicable.

**Table 3:** Epidemiology studies on AMD risk associated with dietary intake and/or serum levels of lutein and zeaxanthin.

Authors (Year)	Study Name	Participants	Follow-Up	Assessment of L/Z	Results
Seddon (1994) [197]	EDCCS	356 AMD patients, 520 controls in USA; aged 55–80 years	-	Dietary L/Z	Highest quintile of L/Z intake, such as spinach and collard greens, strongly associated with reduced risk of late AMD
VandenLangenberg (1998) [201]	Beaver Dam Eye Study	1709 individuals in USA; aged 43–84 years	5 years	Dietary L/Z	No significant association reported between incident large drusen and dietary intake
Mares-Perlman (2001) [194]	NHANES III	8596 individuals in USA; aged ≥40 years	-	Dietary L/Z	Significantly lower risk of pigmentary abnormalities and late AMD in highest L/Z quintiles
Snellen (2002) [198]	-	72 AMD patients, 66 controls in Netherlands; aged ≥60 years	-	Dietary L/Z	Low dietary intake significantly associated with higher risk of neovascular AMD
Cho (2004) [188]	NHS and HPFS	77,562 female and 40,866 male health professionals in USA; aged ≥50 years	18 years; 12 years	Dietary L/Z	No significant association between relative risk of age-related maculopathy and vegetable consumption or carotenoid intake
Van Leeuwen (2005) [200]	The Rotterdam Study	4170 individuals in Netherlands; aged 55–95 years	8 years	Dietary L/Z	No significant association reported between dietary L/Z intake and incident AMD
Moeller (2006) [196]	CAREDS	1787 women in USA; aged 50–79 years	7 years	Dietary L/Z	Protective association among adult women (<75 years) with stable dietary intake and no history of chronic disease
AREDS Research Group (2007) [51]	AREDS	4159 AREDS participants in USA; aged 60–80 years	-	Dietary L/Z	Top quintile of dietary L/Z inversely associated with large drusen, neovascular AMD, and geographic atrophy
Tan (2008) [199]	Blue Mountains Eye Study	2454 individuals in Australia; aged 49–93 years	10.5 years	Dietary L/Z	Greater intake of L/Z saw reduced risk developing soft/reticular drusen and neovascular AMD progression
Cho (2008) [187]	NHS and HPFS	71,494 female and 41,564 male health professionals in USA; aged 50–79 years	18 years; 16 years	Dietary L/Z	A non-linear, inverse association seen among top quintiles of L/Z intake and neovascular AMD in both cohorts
Ho (2011) [16]	The Rotterdam Study	2167 individuals in Netherlands; aged ≥55 years	8 years	Dietary L/Z	Top tertile of L/Z intake significantly reduced incident early AMD in those with greater genetic risk
Wu (2015) [202]	NHS and HPFS	63,443 female and 38,603 male health professionals in USA; aged 50–90 years	26 years; 24 years	Dietary L/Z	Greater consumption of cooked spinach (0.5 cup, >1 serving/wk) inversely associated with intermediate AMD. Late AMD risk significantly lowered by up to 40% with higher L/Z intake
Arslan (2019) [186]	-	100 AMD patients, 100 controls in Turkey; aged ≥50 years	-	Dietary L/Z	Non-significant association observed between serum L/Z
EDCCS Group (1993) [191]	EDCCS	421 AMD patients, 615 controls in USA; aged 55–80 years	-	Serum L/Z	Protective association with greater serum L/Z levels and risk of neovascular AMD
Mares-Perlman (1995) [193]	Beaver Dam Eye Study	167 AMD patients, 167 controls in USA; aged 43–84 years	-	Serum L/Z	No overall association between serum L/Z and risk of late AMD
Gale (2003) [192]	-	380 individuals in Sheffield, United Kingdom; aged ≥60 years	-	Serum L/Z	Serum Z strongly associated with risk of incident early and late AMD
Dasch (2005) [189]	MARS	586 AMD patients, 182 controls in Germany; aged 59–82 years	-	Serum L/Z	No significant association reported between serum L/Z levels
Delcourt (2006) [190]	POLA	640 individuals in Sète, France; aged ≥60 years	-	Serum L/Z	Highest combined serum L/Z has significantly reduced risk
Michikawa (2009) [195]	-	722 individuals in Karabuchi Town of Takasaki City, Japan; aged ≥65 years	-	Serum L/Z	No significant association found between serum L/Z
Zhou (2011) [109]	-	174 AMD patients, 89 controls in China; aged 50–88 years	-	Serum L/Z	Significant inverse association between serum Z and neovascular AMD

Abbreviations: L, lutein; Z, zeaxanthin; AMD, age-related macular degeneration; EDCCS, Eye Disease Case-Control Study; NHANES III, The Third National Health and Nutritional Examination Study; NHS, Nurse’ Health Study; HPFS, Health Professionals Follow-up Study; CAREDS, Carotenoids in Age-Related Eye Disease Study; AREDS, Age-Related Eye Disease Study; MARS, Muenster Aging and Retina Study; POLA, Pathologies Oculaires Liées à l’Âge Study.

**Table 4:** Characteristics of the interventional studies reporting on serum carotenoid levels in AMD.

Authors (Year)	Study	Participants	Duration	Interventions	Serum	Main Findings
Rosenthal (2006) [227]	-	30 patients with intermediate or late AMD; aged 60–91 years in USA	6 months	2.5 mg L; 5 mg L; 10 mg L	L and Z	Mean serum concentrations rose in each dosage group by 2-fold, 2.9-fold and 4-fold, respectively (*p* < 0.001 for all)
Trieschmann (2007) [119]	LUNA	100 patients with AMD; aged (71.5 ± 7.1) years in Germany	6 months	12 mg L and 1 mg Z * (*multivitamin*); placebo	L and Z	Substantial increase in L (4-fold rise; *p* < 0.001) and Z (*p* = 0.007) concentrations
Connolly (2010) [213]	MOST	5 patients with early AMD; aged (72.0 ± 11.0) years in Ireland	2 months	7.3 mg MZ, 3.7 mg L and 0.8 mg Z	L, Z and MZ	Significant time effect between rise in all three carotenoid serum levels (*p* < 0.003 for all)
AREDS2 Research Group (2013) [50]	AREDS2	4203 patients with intermediate or late AMD; aged (73.1 ± 7.7) years in USA	5 years	10 mg L and 2 mg Z * (*multivitamin*); 10 mg L, 2 mg Z and omega-3 fatty acids * (*multivitamin*); “placebo”	L and Z	Total serum L + Z levels increased by 190% to 210% from baseline (*p* < 0.001)
Arnold (2013) [206]	-	20 patients with atrophic AMD; aged (66.0 ± 8.0) years in Germany	4 weeks	10 mg L and 3 mg Z, *in oleaginous kale extract*	L and Z	Statistically significant rise in serum L and serum Z after 4 weeks (*p* < 0.001 for both)
Arnold (2013) [207]	LUTEGA	172 patients with atrophic AMD; aged (69.0 ± 10.0) years in Germany	12 months	10 mg L and 1 mg Z * (*multivitamin*); 20 mg L and 2 mg Z * (*multivitamin*); placebo	L and Z	Beneficial alterations seen in both treatment groups (*p* < 0.05) after one-month and values remained elevated until trial completion
Huang (2013) [219]	-	108 patients with early AMD; aged 50–81 years in China	48 weeks	10 mg L; 20 mg L; 10 mg L and 10 mg Z; placebo	L and Z	Greater increase in serum L and Z with high-dose L (6.23-fold) and L + Z formula (3.11-fold), respectively (*p* < 0.001 for both)
Meagher (2013) [221]	-	27 patients with early AMD; aged (66.0 ± 9.0) years in Ireland	8 weeks	20 mg L, 2 mg Z and 0.3 mg MZ; 10 mg L, 2 mg Z and 10 mg MZ; 3 mg L, 2 mg Z and 17 mg MZ	L, Z and MZ	Serum L and Z increased only with higher-dose L (Groups 1 and 2; *p* < 0.001) while serum MZ increased in all three groups (*p* < 0.01 for all)
Murray (2013) [222]	CLEAR	72 patients with early AMD; aged (70.5 ± 8.7) years in United Kingdom	12 months	10 mg L; placebo	L	Marked increase in serum L (*p* < 0.001) compared to placebo control
Akuffo (2015) [205]	MOST	52 patients with early AMD; aged (66.0 ± 8.0) years in Ireland	3 years	20 mg L and 2 mg Z; 10 mg L, 2 mg Z and 10 mg MZ; 3 mg L, 2 mg Z and 17 mg MZ	L, Z and MZ	Statistically significant time x treatment effect revealed for changes serum L and MZ (*p* < 0.05 for both) concentrations
Huang (2015) [115]	-	112 patients with early AMD; aged (69.1 ± 7.4) years in China	24 months	10 mg L; 20 mg L; 10 mg L and 10 mg Z; placebo	L and Z	Highly significant time x treatment interaction observed for both serum L and Z (*p* < 0.001 for both)
Wolf-Schnurrbusch (2015) [231]	-	79 patients with early/intermediate AMD; aged 55–88 years in Switzerland	6 months	10 mg L and 1 mg Z * (*multivitamin*); 10 mg L, 1 mg Z and omega-3 fatty acids * (*multivitamin*); placebo	L and Z	Increases in serum L and Z (*p* < 0.05) only reported in Group 1 (carotenoid treatment without omega-3 fatty acids in formula)
Akuffo (2017) [204]	CREST	121 patients with early/intermediate AMD; aged (64.7 ± 9.0) years in Ireland	24 months	10 mg L and 2 mg Z * (*multivitamin*); 10 mg L and 10 mg Z * (*multivitamin*); placebo	L, Z and MZ	Remarkable increase in all three serum concentrations (*p* < 0.0005); time x group interaction effect only for serum Z and MZ (*p* < 0.005 for both)
Sawa (2020) [229]	Sakai Lutein Study	39 patients with neovascular AMD; aged (70.7 ± 5.3) years in Japan	6 months	20 mg L and 3 mg Z (*beeswax capsule*); 20 mg L and 3 mg Z (*glycerol capsule*)	L	Serum L increased in both treatment groups at 3- and 6 months (*p* < 0.01 for both)
García-Layana (2021) [217]	-	109 patients with neovascular AMD; aged (77.1 ± 7.6) years in Spain	12 months	10 mg L and 2.6 mg Z * (*multivitamin*); original AREDS formula (*no L/Z*)	L and Z	Substantial increase in serum L and Z (*p* < 0.001 for both) with a large effect size after 12 months (Cohen’s d of ≥0.80 for both)

* Multivitamin treatment containing carotenoids, in combination with other antioxidants. Abbreviations: L, lutein; Z, zeaxanthin; MZ, *meso*-zeaxanthin; AMD, age-related macular degeneration; LUNA, Lutein Nutrition effects (measured by autofluorescence); MOST, meso-zeaxanthin Ocular Supplementation Trial; LUTEGA, Lutein/zeaxanthin and omega-3 supplementation on optical density of AMD patients; CLEAR, Combination of Lutein Effects in the Aging Retina; CREST, Central Retinal Enrichment Supplementation Trials.

**Table 5:** A summary of the eligible randomized clinical trials reporting on AMD.

Authors (Year)	Study	Participants	Duration	Interventions	MPOD	Main Findings
Richer (2004) [225]	LAST	90 patients with atrophic AMD; aged (74.7 ± 7.4) years in USA	12 months	10 mg L; 10 mg L * (*multivitamin*); placebo	HFP	Significant benefit in MPOD (*p* < 0.001), BCVA (*p* < 0.01) and CS at low/middle spatial frequencies (*p* < 0.05 for all)
Bartlett (2007) [209]	-	25 patients with atrophic AMD; aged (69.2 ± 7.8) years in USA	9 months	6 mg L; placebo	-	Non-significant trend towards improvement in CS reported
Cangemi (2007) [212]	TOZAL	37 patients with atrophic AMD; aged (76.3 ± 7.8) years in USA	6 months	8 mg L and 0.4 mg Z * (*multivitamin*)	-	Modest improvements observed in BCVA (*p* = 0.045)
Trieschmann (2007) [119]	LUNA	100 patients with AMD; aged (71.5 ± 7.1) years in Germany	6 months	12 mg L and 1 mg Z * (*multivitamin*);placebo	Fundus AFI	Mean increase of +15.9% in MPOD measured at 0.5° eccentricity (*p* < 0.001) compared to control
Parisi (2008) [223]	CARMIS	27 patients with atrophic AMD; aged (65.5 ± 5.1) years in Italy	12 months	10 mg L + 1 mg Z * (*multivitamin*); placebo	-	Enhanced improvement in central retinal function measures on mfERG (ring 1 and ring 2; *p* < 0.01 for both)
Connolly (2010) [213]	MOST	5 patients with early AMD; aged (72.0 ± 11.0) years in Ireland	2 months	7.3 mg MZ, 3.7 mg L and 0.8 mg Z	cHFP	Significant increase in MPOD measured at 0.25° and 1° eccentricity with respect to time (*p* < 0.05 for all)
Richer (2011) [226]	ZVF	60 patients with early/intermediate AMD; aged (74.9 ± 10.0) years in USA	12 months	8 mg Z; 8 mg and 9 mg L; 9 mg L	HFP	Central (1°) MPOD increased in all three groups (*p* < 0.03 for all); significant improvement in measures of foveal vision greater in Z-only group, while benefits in parafoveal vision were greater in L-only group
Weigert (2011) [120]	LISA	126 patients with early/intermediate AMD; aged (71.6 ± 8.6) years in Austria	6 months	20 mg L for 3 months, then 10 mg L for 3 months; placebo	Reflectometry	Average increase of +27.9% in MPOD (*p* < 0.001); trend toward improvement in BCVA did not reach statistical significance
Ma (2012) [117]	-	108 patients with early AMD; aged 50–81 years in China	48 weeks	10 mg L; 20 mg L; 10 mg L and 10 mg Z; placebo	Fundus AFI	Significant dose-response effect with increased MPOD (*p* < 0.01) positively related to benefits in CS (*p* < 0.05) and central retina function on mfERG (*p* < 0.01)
Piermarocchi (2012) [224]	CARMIS	145 patients with atrophic AMD; aged (72.5 ± 7.0) years in Italy	24 months	10 mg L + 1 mg Z * (*multivitamin*); placebo	-	Reported significant benefits in BCVA and CS at 6-, 12-, and 24 months (*p* < 0.01 for all) compared to placebo
AREDS2 Research Group (2013) [50]	AREDS2	4203 patients with intermediate or late AMD; aged (73.1 ± 7.7) years in USA	5 years	10 mg L and 2 mg Z * (*multivitamin*); 10 mg L, 2 mg Z and omega-3 fatty acids * (*multivitamin*); “placebo”	-	Reduced hazard ratios of 0.82 (95% CI:0.69–0.96; *p* = 0.02) for late AMD and 0.76 (95% CI: 0.64–0.94; *p* = 0.01) for neovascular AMD compared to β-carotene in formulation ^†^
Arnold (2013) [206]	-	20 patients with atrophic AMD; aged (66.0 ± 8.0) years in Germany	4 weeks	10 mg L and 3 mg Z, *in oleaginous kale extract*	Reflectometry	Enhanced augmentation of macular pigment parameters including volume, area and maxOD (*p* < 0.001 for all)
Beatty (2013) [210]	CARMA	433 patients with early AMD; aged (73.9 ± 8.1) years in Ireland	12 months	12 mg L and 0.6 mg Z * (*multivitamin*); placebo	Raman spectroscopy	Statistically significant increase in MPOD with a positive linear trend during trial period (*p* < 0.01 for all)
Berrow (2013) [211]	-	14 patients with early AMD; aged (67.6 ± 8.4) years in UK	40 weeks	12 mg L and 0.6 mg Z * (*multivitamin*); placebo	-	Remarkable benefits in mfERG N1P1 response amplitude densities in ring 3 (*p* = 0.027); no differential changes observed in BCVA and CS
Dawczynski (2013) [215]	LUTEGA	145 patients with atrophic AMD; aged (70.0 ± 10.0) years in Germany	12 months	10 mg L and 1 mg Z * (*multivitamin*); 20 mg L and 2 mg Z * (*multivitamin*); placebo	Reflectometry	Significant improvements observed for MPOD parameters (volume, area, maxOD and mean OD) and BCVA (*p* < 0.001 for all) in both treatment groups
García-Layana (2013) [216]	-	44 patients with early AMD; aged (68.5 ± 8.5) years in Spain	12 months	12 mg L and 0.6 mg Z * (*multivitamin*); placebo	HFP	Considerable rise in MPOD (+0.162 ODU; *p* < 0.01); however, no significant changes seen in BCVA and CS
Murray (2013) [222]	CLEAR	72 patients with early AMD; aged (70.5 ± 8.7) years in United Kingdom	12 months	10 mg L; placebo	cHFP	Highly statistically significant increase in MPOD (+39.5%; *p* < 0.001) when compared to placebo
Sabour-Pickett (2014) [228]	MOST	52 patients with early AMD; aged (66.0 ± 8.0) years in Ireland	12 months	20 mg L and 2 mg Z; 10 mg L, 2 mg Z and 10 mg MZ; 3 mg L, 2 mg Z and 17 mg MZ	cHFP	Robust improvements in MPOD spatial profile observed in those supplemented all three carotenoids in formulation (Group 2, *p* < 0.005; Group 3, *p* < 0.05)
Akuffo (2015) [205]	MOST	52 patients with early AMD; aged (66.0 ± 8.0) years in Ireland	3 years	20 mg L and 2 mg Z; 10 mg L, 2 mg Z and 10 mg MZ; 3 mg L, 2 mg Z and 17 mg MZ	cHFP	Clinically meaningful CS benefits were seen in all three groups (*p* < 0.05 for all): Group 1 (15.15 cpd), Group 2 (1.2-, 6- and 9.6 cpd) and Group 3 (6-, 9.6- and 15.15 cpd)
Huang (2015) [115]	-	112 patients with early AMD; aged (69.1 ± 7.4) years in China	24 months	10 mg L; 20 mg L; 10 mg L and 10 mg Z; placebo	Fundus AFI	Highly significant time x treatment interaction (*p* < 0.001) between changes in MPOD and central retinal function improvements (mfERG and MRS; *p* < 0.05 for both)
Thurnham (2015) [230]	-	32 patients with early AMD; aged (66.0 ± 9.0) years in Ireland	8 weeks	20 mg L, 2 mg Z and 0.3 mg MZ; 10 mg L, 2 mg Z and 10 mg MZ; 3 mg L, 2 mg Z and 17 mg MZ	cHFP	Significant increase in all three groups (*p* < 0.05); Group 2 formulation (10 mg L, 2 mg Z and 10 mg MZ) may offer greater improvement to macular pigment spatial profile
Wolf-Schnurrbusch (2015) [231]	-	79 patients with early/intermediate AMD; aged between 55–88 years in Switzerland	6 months	10 mg L and 1 mg Z * (*multivitamin*); 10 mg L, 1 mg Z and omega-3 fatty acids * (*multivitamin*); placebo	Fundus AFI	Demonstrable benefits in MPOD (*p* < 0.005) and CS scores (*p* < 0.01) observed in Group 1 only (carotenoid treatment without omega-3 fatty acids in formulation)
Akuffo (2017) [204]	CREST	121 patients with early/intermediate AMD; aged (64.7 ± 9.0) years in Ireland	24 months	10 mg L, 2 mg Z and 10 mg MZ * (*AREDS2 multivitamin*); 10 mg L and 10 mg Z * (*AREDS2 multivitamin*)	cHFP	Augmentation of MPOD (*p* < 0.001) with clinically meaningful benefits in visual function (CS and GD under mesopic and photopic conditions, photostress recovery, and mean/max reading speed; *p* < 0.05 for all)
Azar (2017) [208]	-	79 patients with neovascular AMD; aged (75.3 ± 7.6) years in France	8 months	5 mg L and 1 mg Z * (*multivitamin*); placebo	Reflectometry	Non-significant trend toward greater MPOD levels reported in patients with neovascular AMD
Corvi (2017) [214]	-	39 patients with early AMD; aged (78.0 ± 6.5) years in France	3 months	10 mg L and 2 mg Z * (*multivitamin*)	HFP	Significant rise in MPOD only in eyes with reticular pseudodrusen (n = 19; *p* = 0.002) after 3 months
Davey (2020) [53]	-	56 patients with subclinical AMD; aged (68.4 ± 5.3) years in USA	6 months	15 mg L, 10 mg MZ and 2 mg Z * (*Lumega-Z*); 10 mg L and 2 mg Z * (*AREDS-2 multivitamin*); placebo	HFP	Statistically significant CS improvements for Lumega-Z group only (*p* < 0.001) with a positive linear trend with treatment time (*p* < 0.001)
Sawa (2020) [229]	Sakai Lutein Study	39 patients with neovascular AMD; aged (70.7 ± 5.3) years in Japan	6 months	20 mg L and 3 mg Z (*beeswax capsule*); 20 mg L and 3 mg Z (*glycerol capsule*)	Fundus AFI	No significant changes were observed in CS, mesopic glare or MPOD in both treatment groups

* Multivitamin treatment containing carotenoids in combination with other antioxidants; ^†^ Secondary analyses reported in AREDS2 Report No. 3 [203]; Abbreviations: MPOD, macular pigment optical density; L, lutein; Z, zeaxanthin; MZ, *meso*-zeaxanthin; AMD, age-related macular degeneration; LAST, Lutein Antioxidant Supplementation Trial; HFP, heterochromatic flicker photometry; ODU, optical density units; BCVA, best-corrected visual acuity; CS, contrast sensitivity; cpd, cycles per degree; TOZAL, Taurine, Omega-3 fatty acids, Zinc, Antioxidants and Lutein; LUNA, Lutein Nutrition effects measures by Autofluorescence; AFI, autofluorescence imaging; CARMIS, Carotenoids in Age-Related Maculopathy Italian Study; mfERG, multifocal electroretinogram; MOST, meso-zeaxanthin Ocular Supplementation Trial; cHFP, customized HFP; ZVF, Zeaxanthin and Visual Function; LISA, Lutein Intervention Study Austria; AREDS-2, Age-Related Eye Disease Study 2; CARMA, Carotenoids in Age-Related Maculopathy; LUTEGA, Lutein/zeaxanthin and omega-3 supplementation on optical density of AMD patients; CLEAR, Combination of Lutein Effects in the Aging Retina; CREST, Central Retinal Enrichment Supplementation Trials.
